# Piezoelectric and Photoconductive Zinc Oxide–Wood Hybrids Obtained by Atomic Layer Deposition

**DOI:** 10.1021/acsnano.5c03854

**Published:** 2025-04-13

**Authors:** Maximilian Ritter, Krzysztof Maćkosz, Jonas Garemark, Ronny Kürsteiner, Christopher H. Dreimol, Ivo Utke, Ingo Burgert, Guido Panzarasa

**Affiliations:** † Wood Materials Science, Institute for Building Materials, ETH Zürich, 8093 Zürich, Switzerland; ‡ WoodTech, Cellulose & Wood Materials, Empa, 8600 Dübendorf, Switzerland; § Laboratory for Mechanics of Materials & Nanostructures, Empa, 3602 Thun, Switzerland

**Keywords:** atomic layer deposition, inorganic−organic hybrids, photoconductivity, piezoelectricity, cellulose

## Abstract

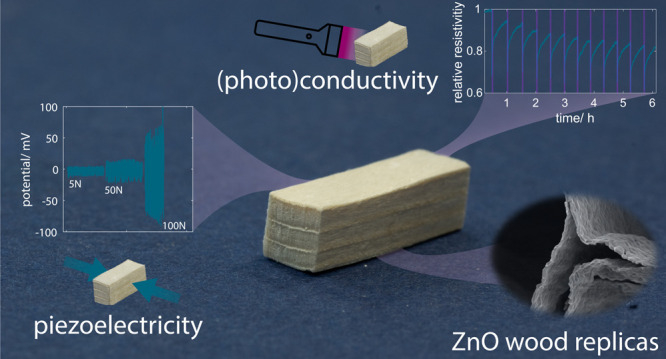

The development of sustainable functional wood-based materials for advanced photonic, optical, and energy-harvesting applications is a topic of great priority and scientific interest. Owing to its inherent piezoactivity and photoconductivity, zinc oxide (ZnO) can be of help for all these applications. While previously used for wood-based piezoelectric nanogenerators, its use for enabling wood with photoconductive properties has not yet been demonstrated. Here, we introduce an innovative method to produce ZnO–wood hybrids based on atomic layer deposition (ALD), a technique so far underrepresented in the field of wood functionalization. By a studied combination of ALD, customized sample geometry, structure-retaining delignification, and careful selection of the drying method, we obtained a homogeneous functionalization of a bulk wood scaffold with layers of nanocrystalline ZnO. This approach allowed us to achieve control over the homogeneity, distribution, and coating thickness of the oxide layer. The micro- and nanostructure of the resulting hybrids were investigated by electron microscopy as well as by X-ray diffraction and scattering. The ZnO–wood hybrids show an anisotropic piezoelectric response due to the natural structure of the wood. Moreover, we demonstrate the use of ZnO-functionalized wood for the fabrication of bulk (photo)conductive wood. Upon irradiation with UV light, a significant decrease in resistivity is observed, which increases again upon removal of UV light. Finally, we used the hybrids to fabricate a ZnO-wood replica by thermal removal of the cellulose scaffold. This treatment leaves behind a detailed inorganic wood replica down to the smallest open accessible features such as micrometer-sized wood pits.

## 1 Introduction

Wood is a state-of-the-art sustainable, CO_2_-storing building material. Enabling wood with new functionalities, such as the ability to generate and conduct electricity, is instrumental in further promoting its use in smart buildings. Piezo- and triboelectric nanogenerators (PENGs and TENGs, respectively) can be made by taking advantage of the intrinsic piezoelectric properties of delignified wood,^1,2^ or using wood as a scaffold for hosting piezo-^3−6^ and triboactive materials.^7,8^ However, while significantly improving the otherwise low electrical output, this latter approach can severely compromise sustainability^7^ since efficient piezoelectric materials (e.g., lead zirconate titanate PZT and barium titanate) often contain rare and toxic metals.^9^

Zinc oxide (ZnO) is a well-known semiconducting material. Thanks to the piezoelectric properties of its wurtzite polymorph,^10^ ZnO can be considered as a more sustainable option for the design of PENGs. The bandgap of ZnO lies in the UV region (∼3.6 eV), making it a photoconductor.^10^ Moreover, the photoconductive behavior of ZnO can be influenced by the presence of certain gases, and its use as a gas sensor has been demonstrated.^11^ Hybrids obtained by introducing ZnO in the bulk of wood have been previously reported,^3,4^ but to the best of our knowledge, they all have been obtained through hydrothermal techniques and characterized mostly for their piezoelectric properties.

Here, we explore atomic layer deposition (ALD) as an alternative technique to achieve a homogeneous coating of open porosity in bulk wood. The ALD of ZnO has already been used, e.g., to obtain homogeneous coatings on hierarchical structures for photovoltaics^12,13^ and three-dimensional metamaterials made of polymers.^14^ We took inspiration from the work of Gregory and co-workers^15^ on the fabrication of ultrathin ZnO coatings on wood through ALD. However, their goal was to improve wood’s durability and thermal conductivity properties, while we were most interested in the possibility of enabling wood with new functionalities, especially photoconductivity and piezoelectricity. ALD is a chemical vapor deposition technique based on a layer-by-layer deposition approach, where a single atomic layer is deposited at a time by self-limiting sequential surface reactions, allowing for high fidelity of the layer thickness and homogeneity, and with all the advantages of a dry method compared to, e.g., hydrothermal ones. By careful tuning of the ALD parameters, a homogeneous ZnO distribution was achieved inside the entire wood scaffold instead of covering only the outside surface. Recently developed ALD scaling laws and models describing the diffusion of gas precursors into open porous media as a function of porosity and area-to-volume ratio enable to adjust process parameters for full penetration and homogeneous coverage.^16,17^ To promote diffusion of the precursors into the bulk of the wood scaffold, we applied structure-retaining delignification followed by two distinct drying approaches. The structural differences obtained by the delignification step as well as the two subsequent drying methods, in turn, influence the distribution of ZnO inside the scaffold. The hydroxyl groups of cellulose are instrumental in achieving a homogeneous ZnO coating, acting as initiation points for the sequential reaction with the diethylzinc precursor.^18^ The micro- and nanostructures of the obtained ZnO–wood hybrid, as well as its piezoelectric and (photo)conductive properties, were analyzed.

## 2 Results and Discussion

### 2.1 Micro- and Nanostructural Characterization

**Figure 1:**
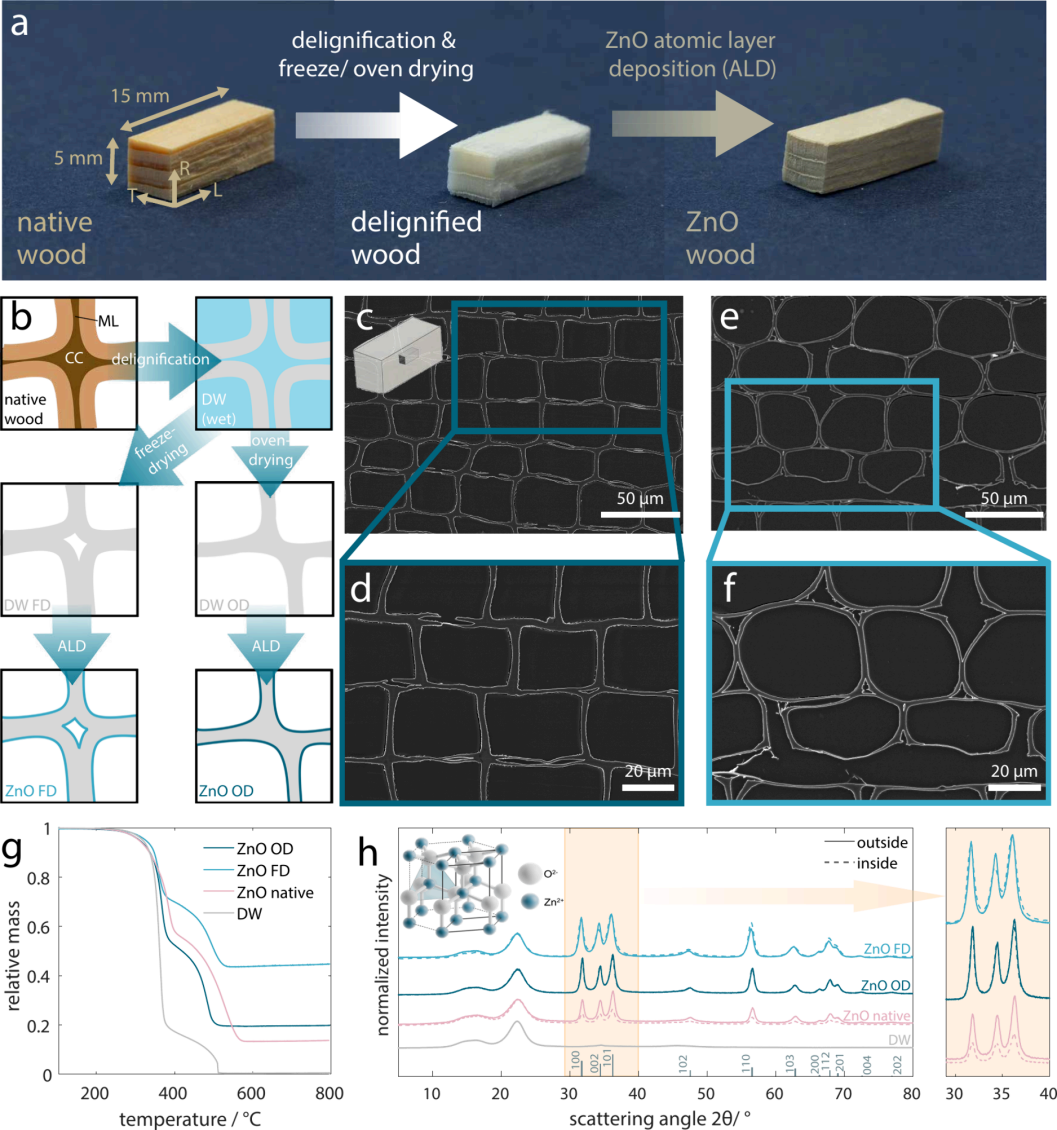
(a) Pictures showing the transition from native to delignified wood and then to a ZnO–wood hybrid. The initial size of the sample was 15 × 5 × 5 mm^3^ (L × R × T). The L-direction corresponds to the fiber direction. (b) Schematic visualization of the delignification process showing a single cell corner (CC) in a cross-sectional view, the removal of the middle lamella (ML) and cell corner, the resulting wood microstructure after oven- and freeze-drying, and finally the areas of ZnO (visualized in light blue) deposition by ALD. Backscattered electron SEM micrographs of *ZnO OD* (c and b) and *ZnO FD* (e and f) cross sections with different magnifications highlighting the structural differences between the two. The inset in (c) highlights the position specimens were taken from. TGA results show the relative mass change as a function of the temperature and the ZnO mass loading once all of the cellulose is burned (g). Diffractograms (normalized to the cellulose 200-peak) collected on the outside and inside of *ZnO OD*, *ZnO FD*, *ZnO native*, and *DW* samples, together with ZnO reference positions (PDF ref. code 00-005-0664), demonstrate the presence of ZnO inside the wood (h).

For our investigations, we chose spruce wood because of its widespread use in Central Europe in the building sector^19^ and its relatively uniform microstructure, but we demonstrate the feasibility of our process also with poplar wood (Figure S1). First, we cut the wood specimens into cuboids with specific dimensions (longitudinal L 15 × radial R 5 × tangential T 5 mm^3^, Figure 1a). This elongated shape along the longitudinal direction is supposed to facilitate the flow of precursors through the wood scaffold during ALD. Then, we performed structure-retaining delignification.^20^ This process removes lignin (and partly hemicelluloses), leaving behind a cellulosic scaffold that retains the structural features of native wood, while opening the lignin-rich middle lamella and the cell corners (Figure 1b).^21^ By opening of the cell corners and middle lamella, an additional accessible surface is generated. During conventional drying (e.g., oven-drying OD), the cell walls collapse, and this newly formed space is lost. However, the open structure can be retained by freeze-drying (FD). We hypothesize that this should lead to the deposition of additional ZnO in the cell corners and potentially inside cell walls (Figure 1b). Furthermore, we hypothesize that the removal of the thickened pit membranes (torus) of the bordered pits between tracheids during delignification^22^ is critical to ensure a homogeneous ALD coating of the delignified wood (DW) scaffold. In the living spruce tree, bordered pits allow for water transport between individual tracheids. When the water pressure drops due to the ingress of air (e.g., when the tree is felled and subsequently wood starts drying), the pressure difference pulls the torus to one side, which results in the sealing of the pit. The closed pits (also termed aspirated pits) isolate the individual cells, impairing fluid transport through the wood. To ensure a successful bulk functionalization by ALD it is therefore crucial to open these aspirated pits, which can be achieved through delignification. To further emphasize the effect of delignification on facilitating the access of ZnO precursors into the bulk, we performed ALD also on native spruce specimens (*ZnO native*). In addition, we prepared ZnO OD specimens with a ZnO layer thickness of only 50 nm (*ZnO OD 50 nm*) to evaluate the precision of the ALD process as well as the effect on the resulting properties. The general process is represented in Figure 1.

**Figure 2:**
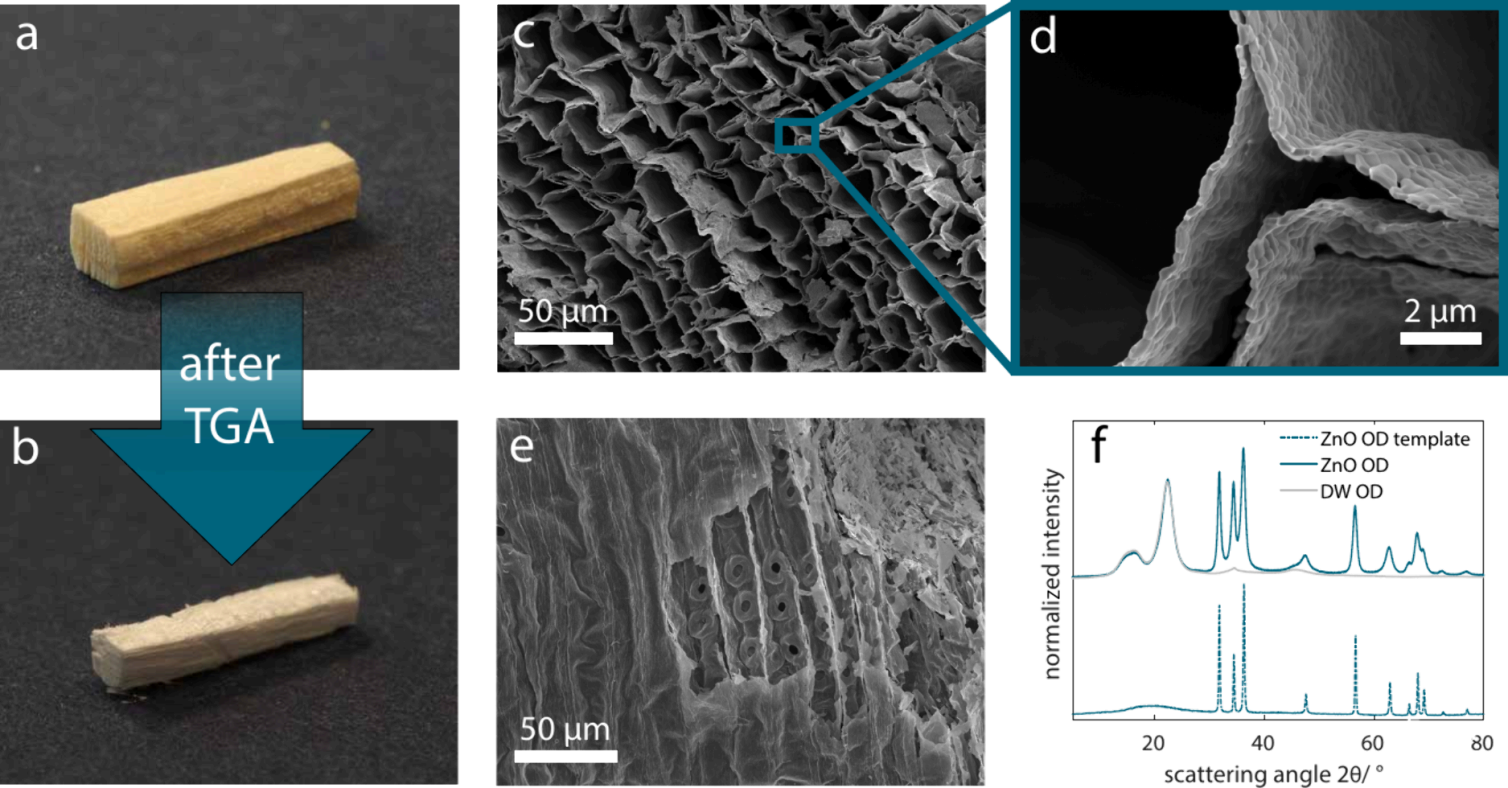
Photo of the *ZnO OD* piece (approximately L 10 × R 2 × T 2 mm^3^) before (a) and after (b) the thermal treatment. (c) SEM micrograph of the replicated wood cross section and (d) a higher magnification view of the retained cell corner. The increase in crystallite size is clearly observable. (e) SEM micrograph of a longitudinally cut replica showing the preservation of the wood pits. (f) X-ray diffractograms of the replica, *ZnO OD*, and *DW OD*. The diffractogram of the replica is missing the cellulose peaks and shows a significant increase in ZnO crystallite size.

For the initial assessment of the resulting ZnO–wood hybrids, we took precautions to ensure proper analysis of the actual specimen core by cutting specimens from the very center of the samples (see the exploded view in Figure 1c). This is necessary to substantiate our claim that by combining structure-retaining delignification and ALD it is possible to homogeneously coat a ZnO layer onto the whole cell walls across the bulk of the specimen. SEM micrographs of cross sections show a homogeneous ZnO coating of the lumina (Figures 1c and 1e) for both the oven-dried and the freeze-dried samples. Higher magnification micrographs (Figures 1d and 1f) also highlight the structural differences between the *ZnO OD* and *ZnO FD*. The micrographs in Figure S2 show that the ZnO coating is homogeneously distributed not only in the earlywood but also in the latewood. As previously discussed, in the case of *ZnO FD* the cell corners remained open, and as hypothesized, they were coated with a ZnO layer. By contrast, as the cell corners collapsed for *ZnO OD*, no coating was visible at this location. It is noteworthy that because of these additional ZnO layers, the drying process has a significant influence on the mass loading of ZnO. For *ZnO OD* the ZnO mass loading (determined by performing TGA measurements in air) was around 20 wt %, while for *ZnO FD* it was almost double (ca. 40 wt %) using the same ALD parameters (Figure 2g). SEM micrographs of *ZnO OD* radial cuts show a homogeneous distribution of ZnO inside the open lumina. Furthermore, they clearly show that the pits were not clogged during the ALD coating and remained open (Figure S3). EDS maps show the presence of Zn in the coating layer (Figure S4), while XRD confirmed the presence of nanocrystalline (crystallite size around 15 nm) ZnO in its wurtzite structure (Figure 2h and Table S1) as well as the presence of cellulose (peaks between 10° and 25° 2θ) from the delignified wood scaffold. The nanocrystalline nature of the ZnO layer is further demonstrated by high-resolution TEM micrographs (Figure S5) and is comparable among all three samples. Diffractograms collected from the surface and the inside of *ZnO OD* and *ZnO FD* specimens confirmed the homogeneous distribution, as the ratio of ZnO to cellulose peaks remains virtually unchanged, indicating a comparable amount of ZnO to cellulose on the outside surface and the inside of the specimen (Figure 2h). In contrast, *ZnO native* showed a less homogeneous ZnO distribution, with significantly lower ZnO peaks (in relation to the cellulose peaks) on the inside than on the outside, indicating less ZnO coated on the inside of the specimen. SEM micrographs further revealed a highly inhomogeneous ZnO coating on the inside of *ZnO native* specimens (Figure S6). Additionally, the overall mass loading of ZnO is lower for *ZnO native*, around 10%.

This preliminary structural characterization indicated the existence of a percolating ZnO distribution, and TGA provided further insight in this regard. Analyzing the samples after TGA, we realized that they kept their overall shape (Figures 2a before and 2b after TGA), even though above 500 °C the delignified wood scaffold should have been completely removed (as is evident from the TGA curves in Figure 1g; images and TGA curves of the remaining samples are shown in Figure S7). This change is already apparent from the white color of the specimens after the thermal treatment. The fact that the shape was retained is a strong indication of the presence of a supporting structure throughout the sample. Further structural analysis revealed that the characteristic microstructural features of wood, including the lumina (Figure 2c), cell corners (Figure 2d), and pits (Figure 2e), were also retained with high fidelity. A significant increase in ZnO crystallite size is visible from the micrographs (especially the high magnification one in Figure 2d), most probably as a result of the high temperatures during the TGA. This increased crystallite size is also apparent from the decreased ZnO peak width in the XRD data in Figure 2f, which lacks the cellulose peaks from the DW scaffold. No orientation can be seen from the wide-angle X-ray diffraction (WAXD) signal either, which also lacks the oriented cellulose reflections from the wood scaffold (Figure S8). However, a strong orientation along the fiber direction can be seen in the small-angle X-ray scattering (SAXS) signal (Figure S8), indicating the presence of some sort of oriented structure on the nanoscale. This is in line with previous reports on templated wood structures, where the SAXS signals also showed pronounced orientation. In one report, this orientation was attributed to the formation of oriented carbon sheets during the pyrolysis of wood in nonoxidizing atmospheres,^23^ but this explanation can be excluded for the present case as the thermal degradation was performed in air. In other reports it was hypothesized that the remaining orientation could be due to a replication of cellulose fibrils and their orientation.^24−26^ To test whether this could also be the case in our system, we functionalized spruce compression wood (that is, wood with a high microfibril angle MFA) and collected its diffraction and scattering signals before and after the thermal degradation of cellulose. Due to the large MFA of the compression wood, the 200-reflections of cellulose were significantly broadened in the functionalized compression wood (*ZnO OD comp.*) (Figures S8b and S8g).^27,28^ Due to the lack of cellulose 200-reflections in the replicas, we used the SAXS signal to determine any potential effect of the MFA on the replicas’ structures, as it was previously shown that both can be equally used for MFA analysis.^27^ The azimuthal profile of *ZnO OD comp.* after the thermal treatment shows no significant differences from the one obtained for *ZnO OD* (Figures S8e, S8f, and S8h). We can therefore safely assume that the MFA is not retained in any form in our wood replicas.

**Figure 3:**
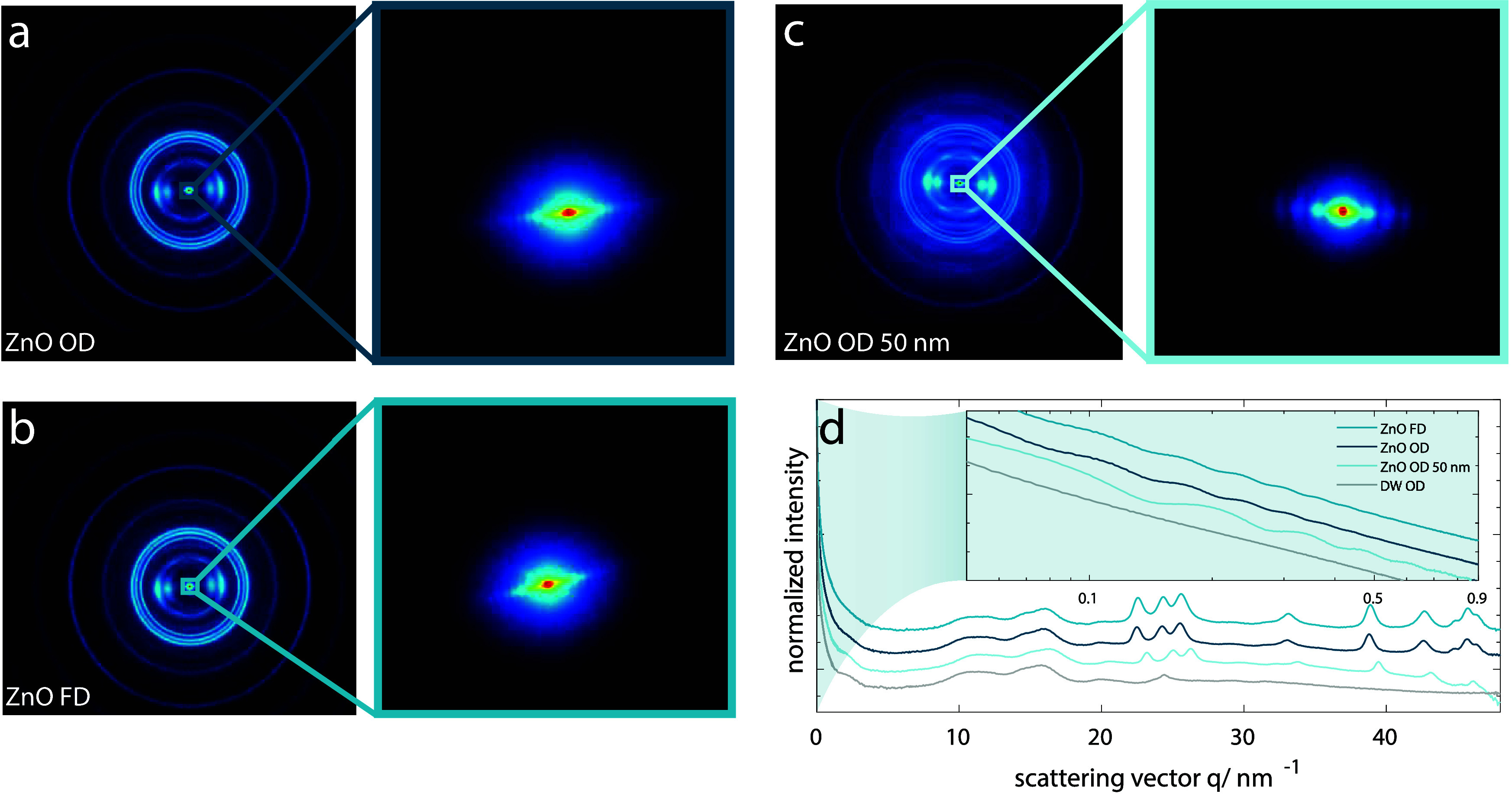
2D WAXD (left) and SAXS (right) patterns for *ZnO OD* (a), *ZnO FD* (b), and *ZnO OD 50 nm* (c) showing the typical anisotropic scattering from the wood in the center and the isotropic ZnO rings around it. (d) 1D integrated scattering and diffraction patterns of the 2D images shown in (a), (b), and (c) and a *DW OD* control with the inset highlighting the features in the small angle regime, which allows the calculation of the ZnO layer thickness.

Next to the functionalization of the entire bulk, one additional advantage of ALD is the possibility of precisely tuning the thickness of the ZnO coating layer. To validate this point for our system we tried to deposit a thinner 50 nm-thick ZnO layer in oven-dried spruce specimens (*ZnO OD 50 nm*). The 2D WAXD patterns indicate the absence of any crystalline orientation inside both 100 and 50 nm-thick ZnO layers in the case of *ZnO OD* samples (Figures 3a–3c, left, respectively). In addition, the reduced intensity of the ZnO peaks (in relation to the cellulose peaks) for the *ZnO OD 50 nm* indicates a lower ZnO content (in line with TGA results presented in Figure S7). The regular scattering features in the 2D SAXS pattern of the specimens (Figures 3a–3c, right, respectively) indicate a very consistent layer thickness across the entire probed volume (beam size 500 × 500 μm^2^, sample thickness between 100 and 200 μm). Already from the 2D WAXD signal it is apparent that the distance between the individual features is larger for the *ZnO OD 50 nm* sample than for *ZnO OD*/*FD* with the intended 100 nm layer thickness, indicating a smaller feature size for *ZnO OD 50 nm*. This difference is considerably more discernible in the integrated 1D scattering data shown in Figure 3d. From the scattering pattern, we calculated the layer thickness by determining the position of the feature minima, which resulted in a layer thickness of 95 nm for *ZnO OD*, 97 nm for *ZnO FD*, and 48 nm for *ZnO OD 50 nm*, respectively. Despite also aiming for a 100 nm ZnO layer in *ZnO native* (data shown in Figure S9), it shows a slightly reduced layer thickness of 92 nm. Additionally, the features are less pronounced, indicating a less homogeneous layer thickness across the probed volume. As previously mentioned, the ZnO wood replicas also feature a strong anisotropic SAXS signal, and from the integrated scattering signal, we can conclude that the layer thickness is retained after the thermal treatment. The calculation, specific values, and a plot highlighting the minima are also shown in Figure S9. These results are confirmed by transmission electron microscopy (TEM) micrographs of the ZnO layer (Figure S5). Overall, these results indicate that by means of ALD, it is possible to precisely control the thickness of the ZnO layer deposited inside the bulk of a delignified wood scaffold.

### 2.2 (Photo)Conductivity Behavior of ZnO–Wood Hybrids

**Figure 4:**
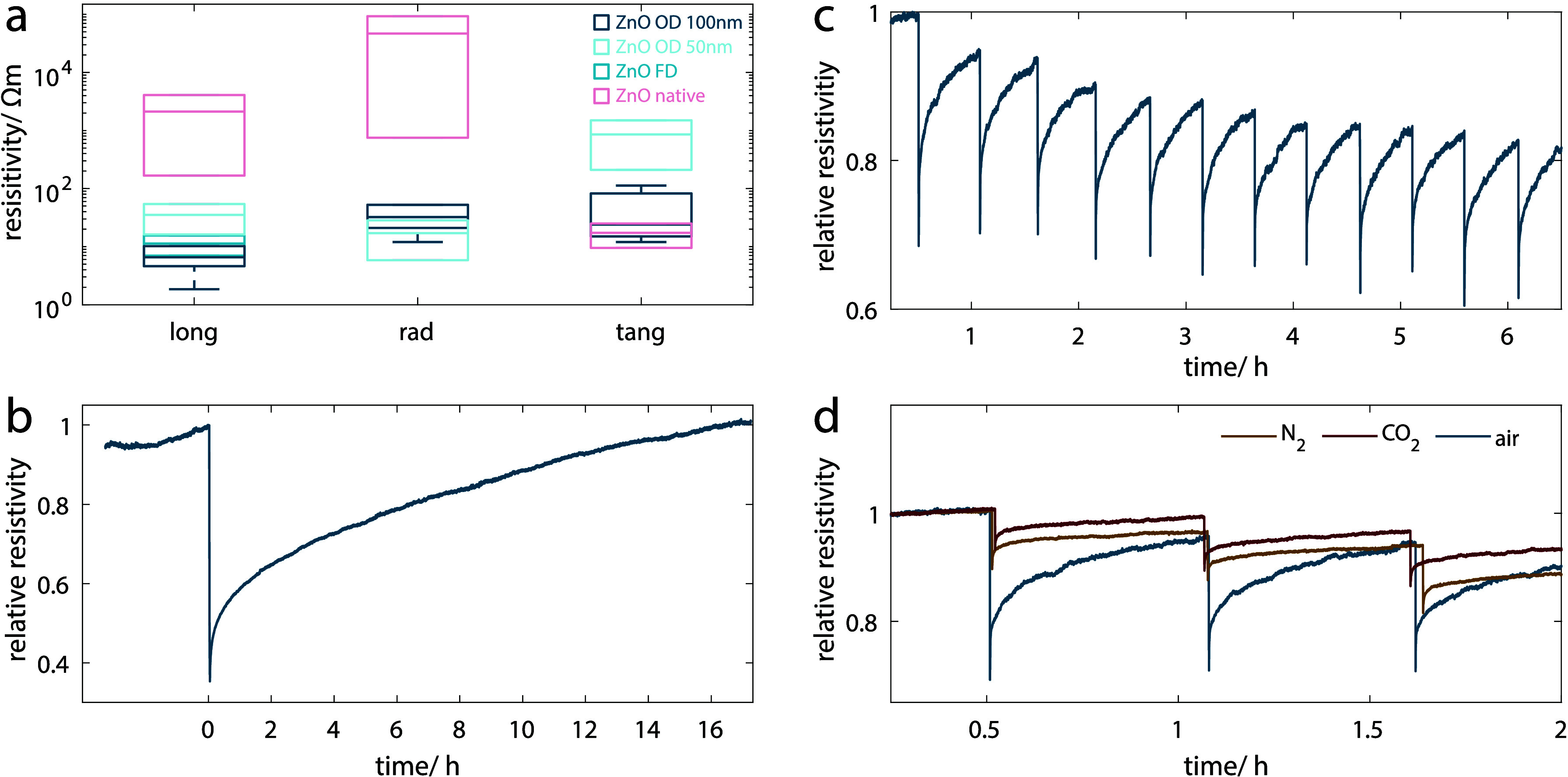
Resistivity values for the different hybrids measured in the longitudinal, radial, and tangential wood directions. While the differences between *ZnO OD*, *ZnO OD 50 nm*, and *ZnO FD* (only measured in the longitudinal direction) are all nonsignificant, the difference in resistivity to *ZnO native* is significant (a). Exposure of *ZnO OD* to 1 min of UV (∼360 nm) led to a significant decrease in resistivity to below 40% of the initial value (b). Continuous and repeated exposure to very short UV pulses led to repeatable cycles (c). Exposing the specimen to different gases (air, N_2_, and CO_2_) when performing the cycling tests shows that the atmosphere had a significant influence on the resistivity decrease (d).

Wood is known to be an electrical insulator, and its resistivity depends strongly on moisture content.^29^ With the introduction of a percolating ZnO network inside the wood scaffold, we expected to measure a significant decrease of resistivity. Moreover, previous reports have shown that bulk electrically conductive wood composites display a highly anisotropic conductivity.^30,31^ For this reason, we performed conductivity measurements along the three main wood directions for all samples (except for *ZnO FD* specimens, which could only be tested in the longitudinal direction due to their fragility). Compared to native wood, the *ZnO OD*, *ZnO FD*, and *ZnO native* hybrids displayed a significantly decreased resistivity, although relatively high compared to values reported in the literature for conductive wood obtained through different approaches.^30−33^ This is not surprising given the semiconducting nature of ZnO and the relatively thin layers obtained by the ALD approach. As illustrated in Figure 4a, no significant differences in the resistivity values can be seen between *ZnO OD*, *ZnO OD 50 nm*, and *ZnO FD* (the latter one only being tested in the longitudinal direction). The only significant difference was found for the three aforementioned samples in relation to *ZnO native*, which had a much higher resistivity. This indicates that the ZnO inside the bulk plays a non-negligible role determining the overall resistivity of the samples. Moreover, while *ZnO OD* showed a relatively pronounced anisotropy in resistivity (6 Ωm in the longitudinal direction, 38 Ωm in the tangential direction, and 46 Ωm in the radial direction), *ZnO OD 50 nm* and *ZnO native* did not show such pronounced trends (*ZnO FD* was only measured along a single direction). The conductivity data are summarized in Table S2. These results further indicate the existence of a percolating ZnO network inside the bulk of the scaffold. It is also noteworthy to mention the comparably low resistivity parallel to the fiber direction, which is hypothesized to result from the coated open bridging pits (Figure S3).

Given the photoconductive nature of ZnO, it is reasonable to assume that the ZnO–wood hybrids would also exhibit this property. Since the bandgap of ZnO is located in the UV region (around 360 nm), we tested the response of *ZnO OD* to UV light exposure. Our initial specimen geometry was not favorable to this kind of test due to its relatively low surface-to-volume ratio. ZnO is a strong UV absorber and has been proposed for the UV-protection of wood.^34^ Therefore, the layers in the bulk of the scaffold could not be reached efficiently by the radiation, resulting in an excessively small reduction in resistivity upon UV exposure. To increase the irradiated surface in relation to the total specimen size, we reduced the specimen size to approximately L 5 × R 2 × T 2 mm^3^. The resistance was always measured along the longitudinal direction. Upon UV irradiation, the resistivity of the specimens immediately started to decrease, and after 1 min of continuous irradiation, it had decreased to 40% of its initial value (Figure 4b). The return to the initial resistivity values after stopping the UV exposure is a substantially slower process. This persistence of photoconductivity is a phenomenon previously described and which has been associated with the adsorption and desorption of oxygen molecules.^35,36^ According to the literature, upon UV irradiation oxygen desorbs from the ZnO surface, increasing the free carriers and thus the conductivity. When the UV exposure is stopped, a slow decrease in conductivity is observed as oxygen molecules readsorb on the surface.^37,38^ For ZnO–wood hybrids, it took around 17 h for the resistivity to return to its initial value after just 1 min of UV irradiation. We observed that after removing the UV source there was an initially fast resistivity increase which flattened toward reaching the initial preirradiation value. The resulting shape of the curve is in accordance with previously reported ones.^35,36^ Due to the higher responsive behavior in the initial phase, we decided to run cycles with both shorter illumination (approximately 1 s) and recovery times (30 min), thus obtaining relatively stable cycles over more than 6 h (Figure 4c). However, this led to a slight drift in the resistivity value immediately after illumination. This drift originates from the inability of resistivity to recover its initial value prior to illumination in the given time frame. To obtain more stable cycles, either shorter illumination times or longer recovery times would be needed. Similar cycles can be achieved by measuring the resistivity changes of a specimen exposed to ambient day–night cycles for about 2 weeks (Figure S10). The increase and decrease in conductivity clearly match the day–night cycle, with the resistivity decreasing during the day (sun and artificial lights) and again increasing during the night. Both cases clearly indicate the possible applicability of the ZnO–wood hybrids as photosensors, especially due to their rapid initial response to direct UV exposure but also their response to changes in ambient light conditions. Moreover, the naturally high specific surface area (SSA) of wood is well preserved in the ZnO–wood hybrids (e.g., *ZnO OD* has a SSA of 122 m^2^/g). The dynamic vapor sorption and desorption (DVS) curves and the SSA values are summarized in Figure S11 and Table S3 and are in line with previous reports on SSA of DW.^39^ We investigated the effect of different atmospheres on the photoconductive response by performing experiments in a quartz glass tube under a controlled atmosphere. In addition to 65% RH air, we tested N_2_ and CO_2_. Both latter atmospheres had a significant influence on the response of the ZnO–wood hybrids to UV illumination, as with N_2_ and CO_2_ the resistivity decreased significantly less upon UV irradiation and the subsequent recovery was less pronounced than with air (Figure 4d).

### 2.3 Piezoelectric Behavior of ZnO–Wood Hybrids

**Figure 5:**
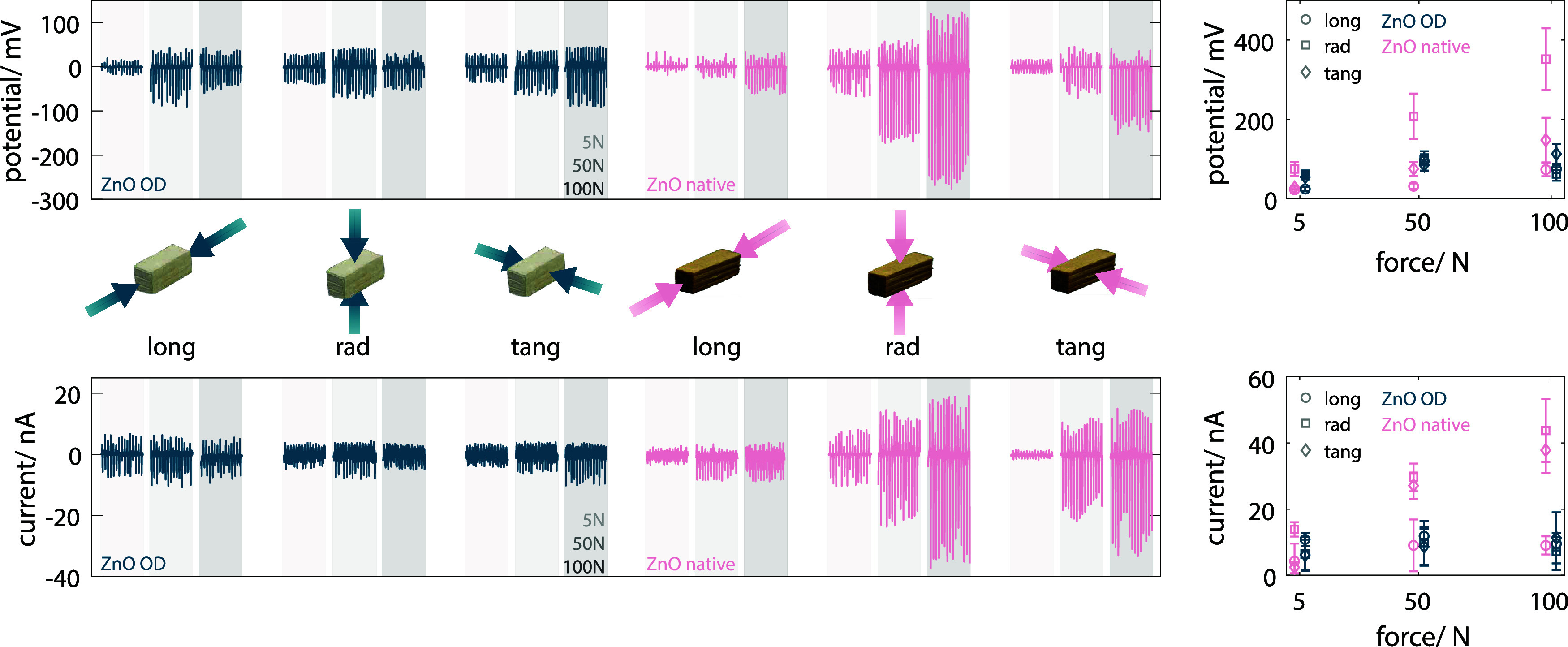
Piezoelectric response (potential at the top and current at the bottom) of *ZnO OD* (dark blue) and *ZnO native* (pink) to the application of 5, 50, and 100 N load. Here, representative 10 s (equivalent to 15 cycles) of measured response are shown, as well as the average peak–peak potential/current of the shown cycles (right).

The wurtzite crystal structure of ZnO is known to be piezoelectric. Indeed, the use of ZnO–wood hybrids, obtained by hydrothermal methods, as piezoelectric nanogenerators has previously been reported.^3,4^ To investigate the piezoelectric behavior of our samples, we attached electrodes and applied loads of 5, 50, and 100 N in the three main wood directions (longitudinal, radial, and tangential). *ZnO FD* samples were not investigated further due to their fragility (already upon applying the lowest 5 N load, the specimens disassembled along the fiber direction). The explanation could be the additional ZnO layer inside the cell corner reducing the interaction between individual tracheids. Therefore, Figure 5 summarizes only the piezoelectric output (voltage and current) of *ZnO OD* and *ZnO native* samples, showing representative 10 s timeframes from 60 s-long measurements (full data are shown in Figure S12). For the initial test with a 5 N load on *ZnO OD*, we obtained a piezoelectric response around 10 mV and 5 nA, with slightly higher values for the radial and tangential directions compared to the longitudinal direction. Unexpectedly, increasing the load from 5 to 50 N and finally 100 N did not significantly increase the piezoelectric response. These results are not in line with other reports in the literature, where an increased load yielded a higher piezoelectric response. We then tested *ZnO native* and found that while the piezoelectric response at 5 N was comparable to the results obtained for *ZnO OD*, an increased load resulted in an increased piezoelectric response for the radial and tangential loading direction. The highest piezoelectric response (ca. 0.4 V and 40 nA) is reached by applying a 100 N load in the radial direction. Given the size of the specimens (15 × 5 × 5 mm^3^), an efficient stress transfer from the outside surface, where the load is applied, to the bulk, where most of the ZnO is located, seems to play a crucial role in the enhanced piezoelectric effect. In the radial and tangential directions, native wood is stiffer than delignified wood. This could explain why the difference between *ZnO native* and *ZnO OD* is much more pronounced in the radial and tangential directions. Additionally, the distribution of ZnO inside the *ZnO native* specimens is rather inhomogeneous, with the regions closer to the surface having a higher ZnO loading (Figure 1h). Nevertheless, both *ZnO native* and *ZnO OD* samples have comparable loadings of ZnO close to the surface (see Figure 1h). According to Ram and co-workers,^3^ in ZnO–wood hybrids the main contribution to the piezoelectric response comes from the outer layers and surface. This could help explain why the total lower ZnO loading does not significantly influence the piezoelectric response of *ZnO native* samples compared to *ZnO OD* ones. Additionally, a strong negative correlation between piezoelectric response and conductivity (that is, the higher the conductivity the lower the piezoelectric response) has been shown for ZnO.^40^ Therefore, the piezoelectric response of *ZnO OD* should be negatively affected by its lower resistivity compared to that of *ZnO native*. Thus, we hypothesize that the reason for the reduced piezoelectric response of the ZnO OD could be due to a complex correlation of these two effects. The piezoelectric response of *ZnO OD 50 nm*, shown in Figure S13, was comparable to that of *ZnO OD* with a 100 nm ZnO layer thickness.

## 3 Conclusion

We report bulk wood functionalization using ALD to achieve a ZnO–wood hybrid with both photoconductive and piezoelectric properties. A detailed micro- and nanostructural study showed the effects of delignification and different drying methods on the overall ZnO distribution inside the (delignified) wood scaffold. Additionally, the ability of the ALD process to allow precise tuning of the ZnO layer thickness was analyzed by successfully depositing both 100 and 50 nm-thick layers. The effect that these parameters have on the final properties of our material, especially piezoelectricity and photoconductivity, was subsequently analyzed. Moreover, we found that this approach can be used to make ZnO replicas of wood scaffolds with remarkable detail and fidelity, which, thanks to the combination of porosity (from the wood scaffold) and photoactivity (from ZnO), could find application, e.g., as in-flow photoreactors.

## 4 Materials and Methods

### 4.1 Delignification

Structure-retaining delignification was performed by placing spruce wood (*Picea abies*) specimens between sheets of stainless-steel mesh, submerging them in an equal-volume mixture of hydrogen peroxide (>30% w/v in water, Fisher Chemicals) and glacial acetic acid (Merck), and leaving them to soak overnight.^20^ After soaking, the delignification solution was heated to 80 °C and specimens were kept inside for 5 h. After this time, the specimens were removed and washed with deionized water until the washing solution reached a neutral pH (approximately pH 7). Oven-dried (OD) specimens were then first solvent exchanged from the neutral washing water to a 50:50 ethanol:water mixture, then to 100% ethanol, further to a 50:50 ethanol:acetone, and finally 100% acetone, before being dried inside an oven at 65 °C. Freeze-dried (FD) samples were frozen from the washing water and then freeze-dried using a Christ Alpha 1-2 LDplus freeze drier.

### 4.2 ALD

ZnO ALD was performed in a customized ALD reactor at 150 °C using diethylzinc and deionized water as reactants. The reactants were kept at room temperature. The porous structures were uniformly coated by 500 ALD cycles (for 100 nm thick ZnO layers) of diethylzinc (Dockweiler Chemicals) and H_2_O with precursor opening, exposure, and argon purge times of 200 ms, 20 s, and 125 s, respectively. The growth per cycle was approximately 1 Å. The *ZnO OD 50 nm* specimens with a 50 nm ZnO layer were coated by 250 ALD cycles.

### 4.3 Small-Angle and Wide-Angle X-ray Scattering (SAXS and WAXS)

SAXS and WAXS measurements were performed using an in-house SAXS machine (Xenocs Xeuss 3.0) equipped with a Cu Kα X-ray source (GeniX 3D; beam size 0.5 × 0.5 mm^2^). The specimens were placed inside the sample chamber with the fiber axis aligned perpendicular to the beam. Native and delignified wood, as well as ZnO hybrid samples, were mounted directly on the Xenocs solid sample holder, whereas the *ZnO OD* replica was first placed inside a glass mark tube (Hilgenberg, 0.7 mm outer diameter and 0.01 mm wall thickness). The 2D scattering signal was recorded under vacuum on a Dectris EIGER2 1 M detector (75 × 75 μm^2^ pixel size). The WAXS data was recorded at a sample-to-detector distance (SDD) of 50 mm (distance calibrated using a LaB_6_ standard), and to extend the *q* range, we used the virtual detector setting in combination with the line eraser mode, collecting 9 × 2 images at different detector positions, which were then stitched together to allow covering a *q*-range (scattering vector *q* = 4π sin(θ)/λ) from around 1 to 47 nm^–1^. SAXS data were recorded at an SDD of 1300 mm (calibrated using a silver behenate standard) with the line eraser function (collecting two images at slightly shifted detector positions to allow a seamless *q*-range from 0.03 to 1.4 nm^–1^). The collected 2D data were azimuthally integrated to obtain the 1D scattering signals. For the MFA analysis, we obtained azimuthal profiles. For the WAXD signal we integrated a 0.4 Å^–1^ wide band around the cellulose 200-peak (1.6 ± 0.2 Å^–1^). For the SAXS signal, the azimuthal profile was obtained from 0.01 to 0.06 Å^–1^. All data handling was performed using the Xenocs XSACT software.

### 4.4 X-ray Diffraction (XRD)

XRD experiments were performed using a Panalytical X’Pert PRO MPD diffractometer and Cu Kα1 radiation (Cu Kα X-ray source and a Ge monochromator). The data was collected in Bragg–Brentano geometry between 5° and 80° 2θ with a step size of 0.03° and a total collection time of 120 min. The specimens were placed on a silicon zero-background holder with their L–T surface facing upward and placed inside the machine. During the data collection, the specimen was rotated at 15 rpm. Reference pattern was taken from the PDF5 database^41^ with reference code 00-005-0664.

### 4.5 SEM and EDS

Scanning electron microscopy (SEM) was performed on a Hitachi SU5000 instrument. The specimens were dried at 103 °C for 12 h and embedded in Spurr low viscosity embedding resin (Sigma-Aldrich). Cross sections were prepared on a Leica EM UC7 ultramicrotome equipped with a 45° diamond knife (Diatome) and coated with 5 nm of carbon by using a Safematic CCU-010 carbon coater. Backscattered electron micrographs were acquired under high vacuum at a 10 kV acceleration voltage. Elemental content distribution mapping was performed by energy dispersive X-ray spectroscopy (EDS) spectrum imaging with an Oxford Ultim Max 100 EDS system. The EDS spectrum was acquired by using 3 kV acceleration voltage with a spectral resolution of 5 eV per channel and an acquisition time of 20 min.

### 4.6 TEM

Specimens for analytical (scanning) transmission electron microscopy ((S)TEM) were dried in extra dry ethanol (Sigma-Aldrich) and embedded in Spurr low viscosity embedding resin (Sigma-Aldrich). Ultrathin sections (∼100 nm) were prepared on an ultramicrotome (Ultracut E, Reichert-Jung) using a diamond knife (Diatome) and flattened using chloroform vapor. The ultrathin sections were deposited on gold grids with an ∼5 nm continuous carbon film. The analysis was performed on an FEI Talos F200X operated at an acceleration voltage of 200 kV. Elemental content distribution mapping was carried out by energy-dispersive X-ray spectroscopy (EDS) STEM spectrum imaging with a windowless Super-X EDS system. The EDS spectra were acquired up to 20 keV with a spectral resolution of 10 eV per channel and an acquisition time of 20 min.

### 4.7 DVS

Water adsorption was characterized by dynamic vapor sorption (DVS; Model Advantage ET 1, Surface Measurement Systems, Ltd., London, UK). Samples were preconditioned at 65 °C for 720 min to outgas any preadsorbed water under a nitrogen atmosphere (N5.0 grade). The samples were then exposed to ascending *p*/*p*_0_ steps of 0, 0.05, 0.10, 0.15, 0.20, 0.25, 0.30, 0.40, 0.50, 0.60, 0.70, 0.75, 0.80, 0.85, 0.90, 0.95, and 0.98 for adsorption and then descending in the same manner for desorption. Stop criteria (reaching quasi-equilibrium) for each RH step was defined as a mass change rate below 0.0003% min^–1^ over a period of 10 min or otherwise a maximum period of 1000 min. During an adsorption experiment, the temperature was set to 25 °C. To regulate the RH steps, we used humidified high-purity N_2_ (5.0 grade) as a carrier gas (constant flow rate of 200 mL min^–1^).

Besides the characterization of the hygroscopicity of individual samples, adsorption isotherms were used to estimate the (specific) surface areas based on the mass increase in the RH range between 15% and 35% RH in accordance with previous analysis.^39^

### 4.8 TGA

TGA was performed under artificial air (PanGas, 20% O_2_ 80% N_2_, air flow 50 mL min^–1^) flow using a platinum pan and a TA TGA Q50. A small piece of around 3–5 mg was cut from the center of the specimen (unless stated otherwise) and inserted into the machine. First, specimens were fully dried by equilibrating them at 103 °C for 30 min. Once equilibrated, we started heating at a constant rate of 10 °C min^–1^ until 800 °C was reached. Given that no changes in mass were detected above 500 °C, we assumed that the organic phase had disappeared at this point. The relative mass (calculated in relation to the dry mass at 103 °C) at 800 °C was used to estimate the ZnO mass loading.

### 4.9 Piezoelectricity Measurements

Electrodes were attached to the ZnO–wood hybrids (on the respective faces depending on the direction of testing to collect HF01-48) to apply a periodic force, measured with a 500 N load cell (Honeywell). The output was measured using a Keithley DMM6500 6 1/2 DIGIT MULTIMETER.

### 4.10 Conductivity Measurements

Electrodes were attached to the ZnO–wood hybrids (on the respective faces depending on the direction of testing) to measure the conductivity. To ensure a proper contact between the electrode and the hybrids, we applied a constant pressure of 10 N to the contact area inside a modified micromechanical testing setup (equipped with a Honeywell 50 N load cell). The conductivity was measured using a Keithley DMM6500 6 1/2 DIGIT MULTIMETER. The resistivity was calculated based on the total sample size (so the L 15 × R 5 × T 5 mm^3^) and was not calculated based on the expected ZnO layer area.

### 4.11 Photoconductivity Measurements

Electrodes were attached to the ZnO–wood hybrids (on the respective faces depending on the direction of testing) to measure the conductivity. The conductivity was measured using a Keithley DMM6500 6 1/2 DIGIT MULTIMETER. The resistivity was calculated based on the reduced sample size (L 5 × R 2 × T 2 mm^3^) and was not calculated based on the expected ZnO layer area. UV illumination was performed using a UV torch (Walther UV5, 5W UV-LED with max 425 lm).

## Supporting Information Available

Additional data (tables and figures) on the ZnO ALD functionalization of poplar, additional crystallographic data (crystallite sizes), EDS maps, (HR)TEM micrographs of the ZnO layer, TGA curves of all samples, MFA analysis of the ZnO–wood hybrids and ZnO wood replicas, detailed layer thickness determination, additional (photo)conductivity data, and the DVS sorption and desorption curves (PDF)
