# Supplementary material for: Piezoelectric and Photoconductive Zinc Oxide–Wood Hybrids Obtained by Atomic Layer Deposition
Source: ACS Nano. 2025 Apr 13;19(15):15161–70. doi: 10.1021/acsnano.5c03854 (PMC12020421; doi:10.1021/acsnano.5c03854)
Supplement: Supplementary file 1 — nn5c03854_si_001.pdf [file nn5c03854_si_001.pdf]

SUPPORTING INFORMATION

Piezoelectric and Photoconductive Zinc Oxide-Wood Hybrids Obtained by Atomic Layer Deposition

Maximilian Ritter[1,2*], Krzysztof Mackosz[3], Jonas Garemark[1,2], Ronny Kürsteiner[1,2], Christopher H. Dreimol[1,2], Ivo Utke[3], Ingo Burgert[1,2], Guido Panzarasa[1*]

1 Wood Materials Science, Institute for Building Materials, ETH Zürich, 8093 Zürich, Switzerland

2 WoodTech, Cellulose & Wood Materials, Empa, 8600 Dübendorf, Switzerland

3 Laboratory for Mechanics of Materials & Nanostructures, Empa, 3602 Thun, Switzerland

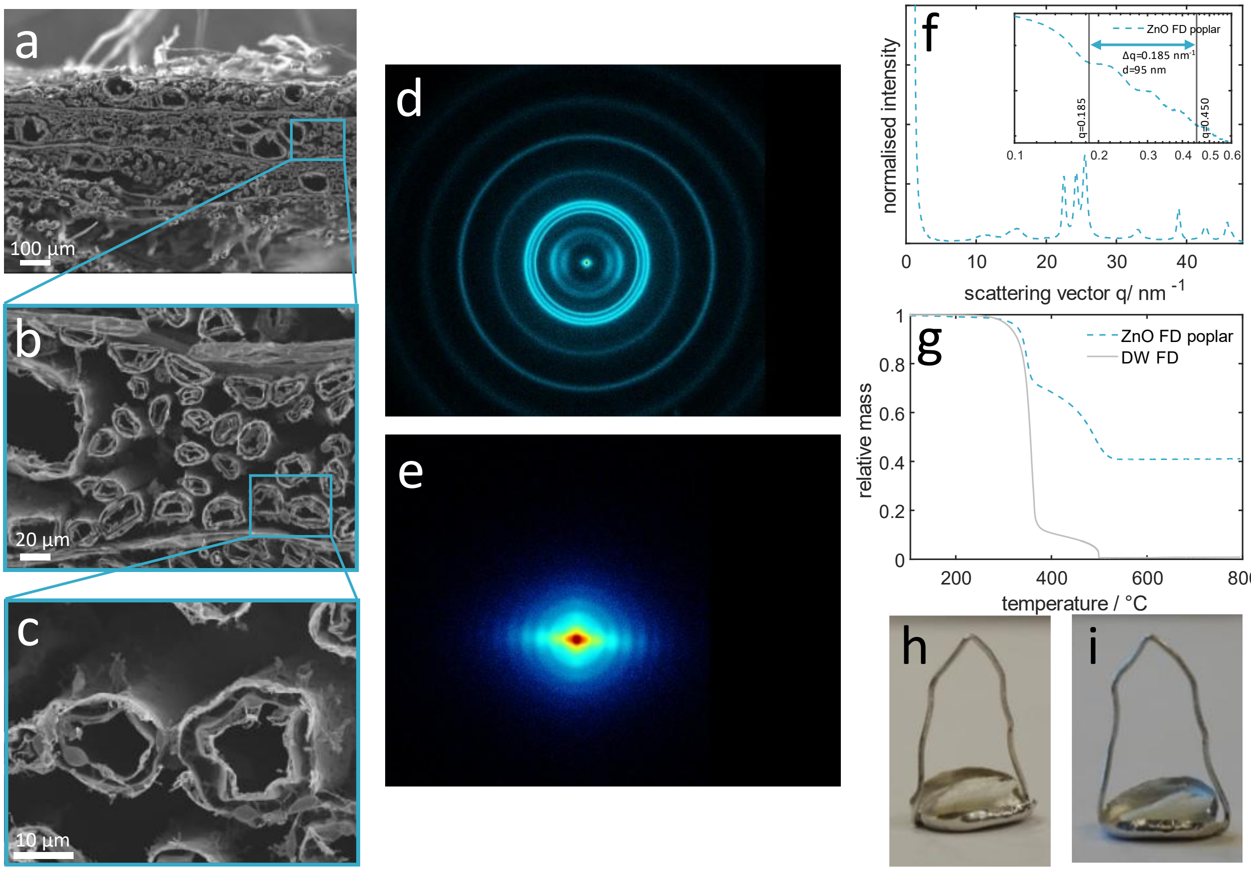

Figure S1. ZnO-wood hybrids using delignified and freeze-dried poplar wood (*ZnO FD poplar*). (**a-c**) Micrographs of the hybrids with different magnifications. (**d**) 2D WAXS signal with the characteristic anisotropic cellulose signal and the isotropic ZnO signal. (**e**) Highly anisotropic SAXS signal. (**f**) Integrated WAXS and SAXS (*insert*) signal. The WAXS diffractogram clearly shows the cellulose and ZnO peaks. From the features of the SAXS signal we calculated the layer thickness, indicating a layer thickness of approx. 95 nm. (**g**) TGA curves of *ZnO FD poplar* and *DW FD*, indicating a ZnO mass loading of around 40%. Pictures of the specimen before (**h**) and after (**i**) the thermal treatment.

Table S1. Crystallite Size of ZnO layer calculated from the FWHM of the respective ZnO peaks in *ZnO OD*, *ZnO FD* and *ZnO native*. Crystallite size was calculated using Sherrer's Equation.

**Peak index**		**100**	**002**	**101**	**102**	**110**	**103**	**200**	**112**	**201**
Crystallite Size / Å	*ZnO FD*	181	117	114	61	135	93	118	127	121
	ZnO OD	161	145	127	67	130	113	121	116	171
	ZnO native	200	161	148	81	167	110	123	171	177

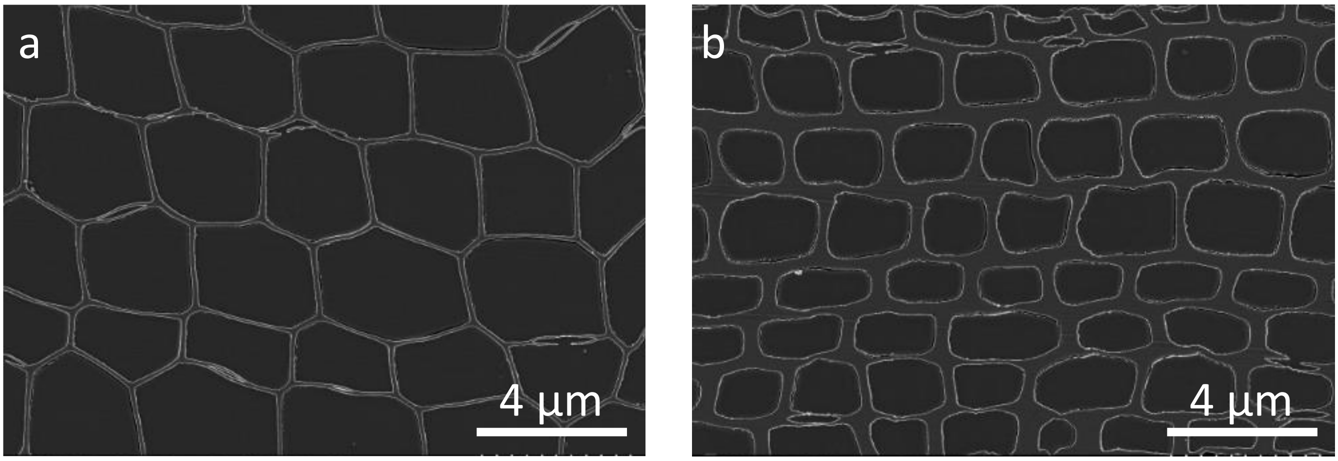

Figure S2. Backscatter SEM micrographs of *ZnO OD* show the homogeneous ZnO distribution, both in the early (**a**) and late wood (**b**).

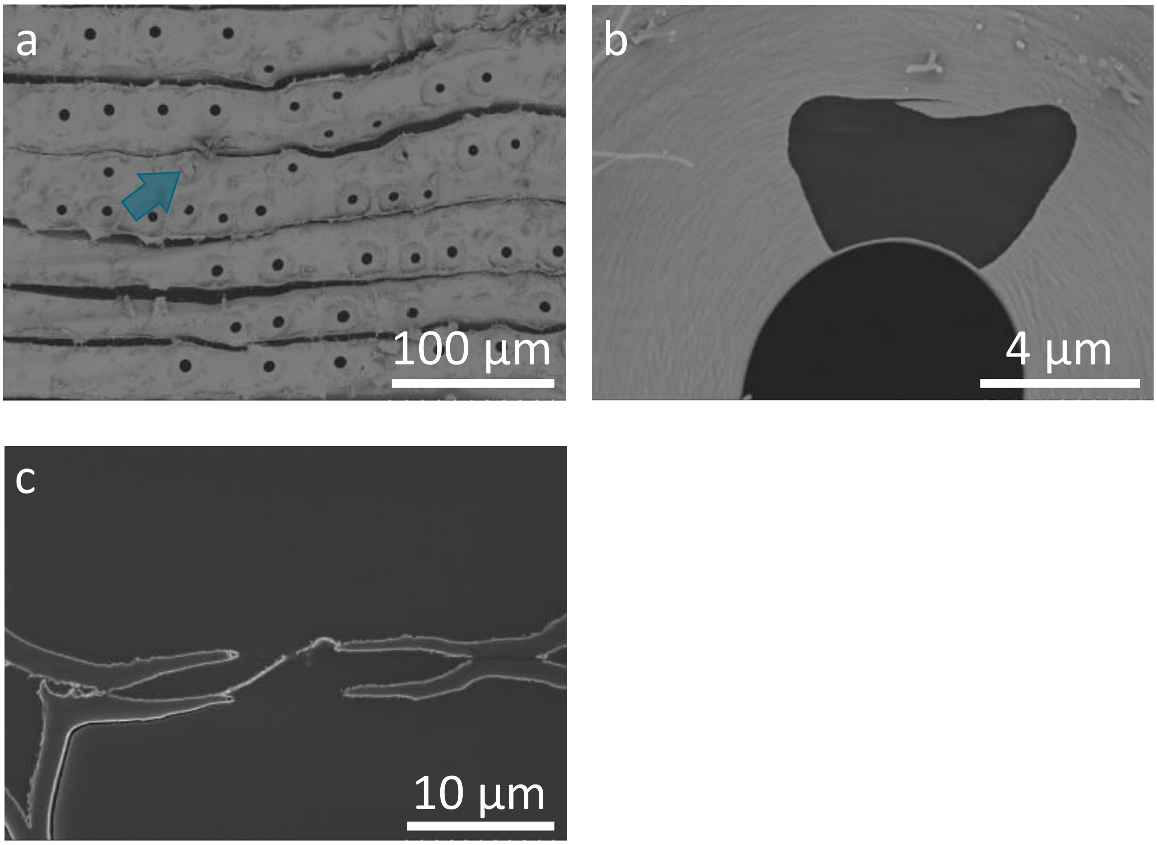

Figure S3. Backscatter SEM micrographs of a radial *ZnO OD* cut showing the homogeneous distribution of ZnO inside the lumen and the open pits. The arrow points at one pit that remained closed. (**a**) Higher magnification micrograph of a pit shows a small area where a part of the ZnO layer was removed. (**b**) Cross sectional view of a fully ZnO coated pit, with a broken ZnO coated torus. (**c**)

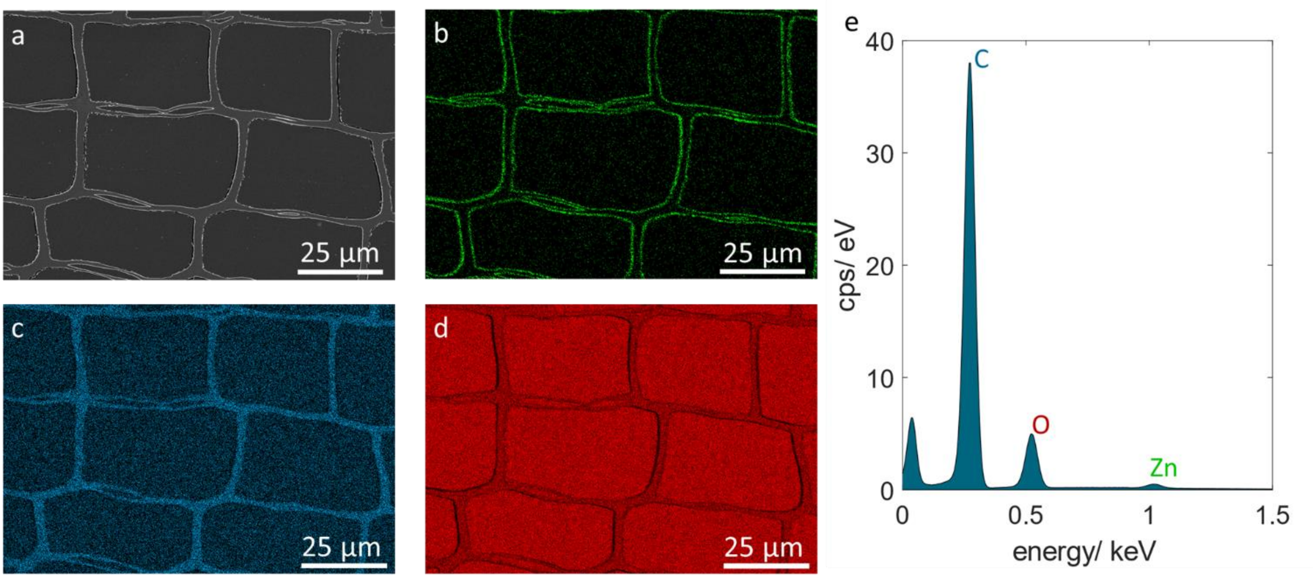

Figure S4. SEM micrograph of *ZnO OD* (**a**) and corresponding EDS maps of Zn (**b**), O (**c**) and C (**d**). The corresponding spectrum is shown in (**e**), where C is C Kα1,2, O is O Kα1 and Zn is Zn Lα1,2. EDS signal from the lumen is from the epoxy used for sample embedding.

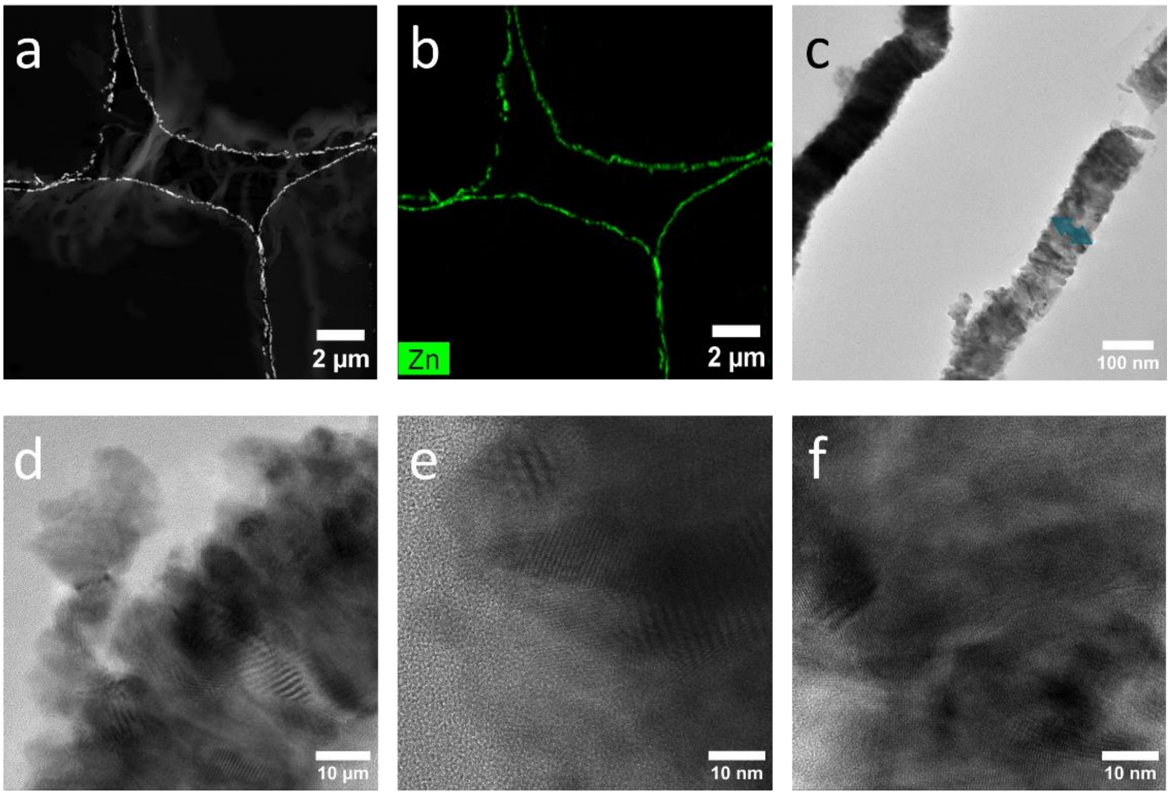

Figure S5. (HR)TEM micrographs of the ZnO layer of *ZnO OD 100 nm*. TEM micrograph and corresponding Zn EDS map (**a**, **b**). Higher magnification of the ZnO layer, arrow indicates 100 nm (**c**). HRTEM micrographs showing the individual nano crystallites, which are in line with results obtained from X-ray diffraction (**d**, **e**, **f**).

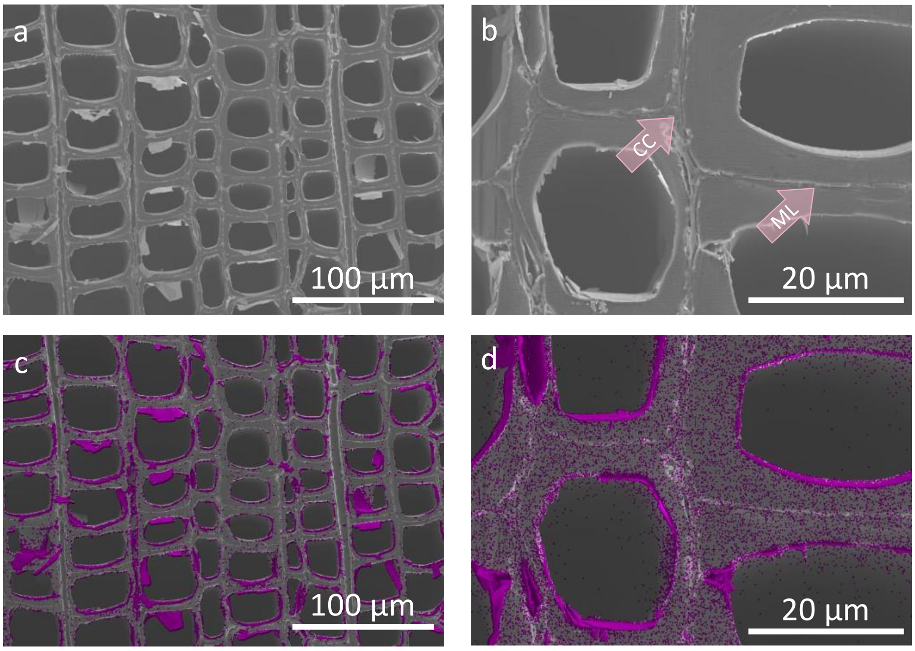

Figure S6. SEM micrographs of *ZnO native* with the ZnO coating clearly visible (**a**, **b**) and corresponding EDS maps indicating the position of Zn inside the specimen (**c**, **d**) indicating the rather inhomogeneous distribution. The cell corner (CC) and middle lamellar (ML) – still present as it is native wood – are highlighted in (**b**).

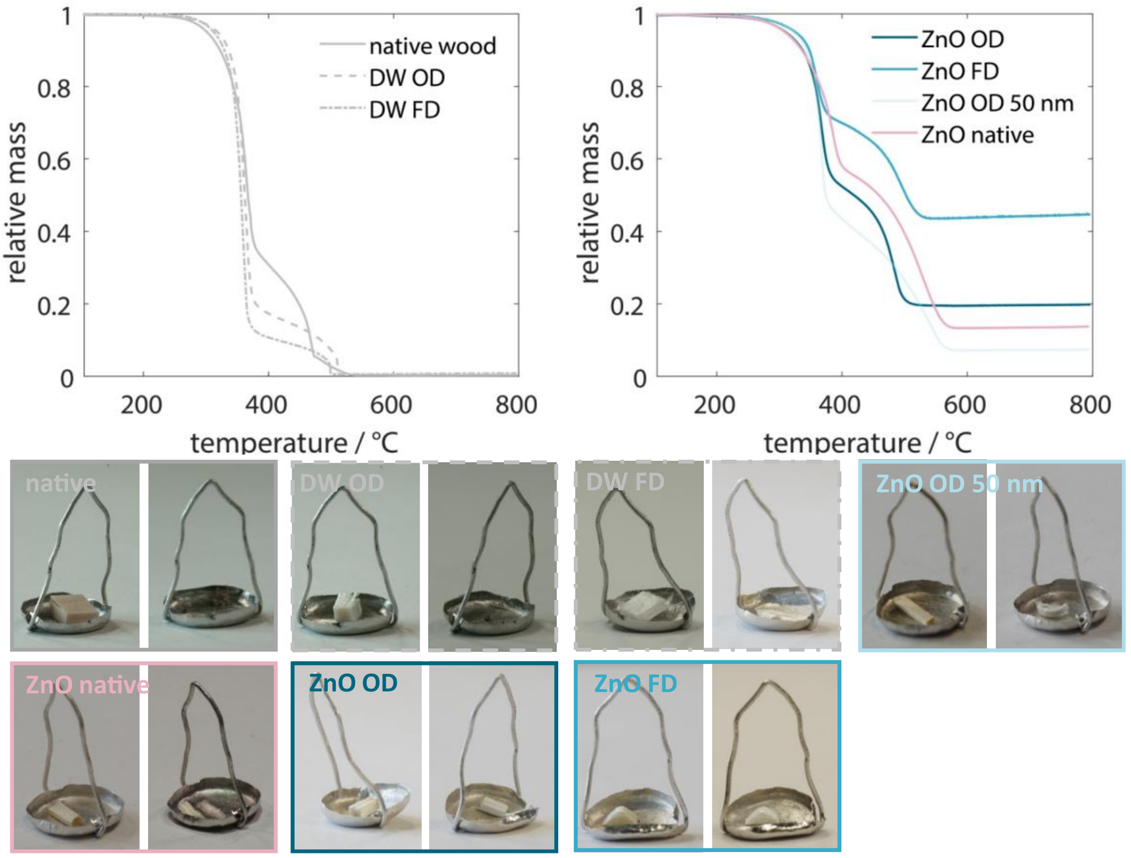

Figure S7. TGA curves (*top*) and photographs (*bottom*) of the obtained replicas for all different ZnO-wood hybrids, native and delignified wood.

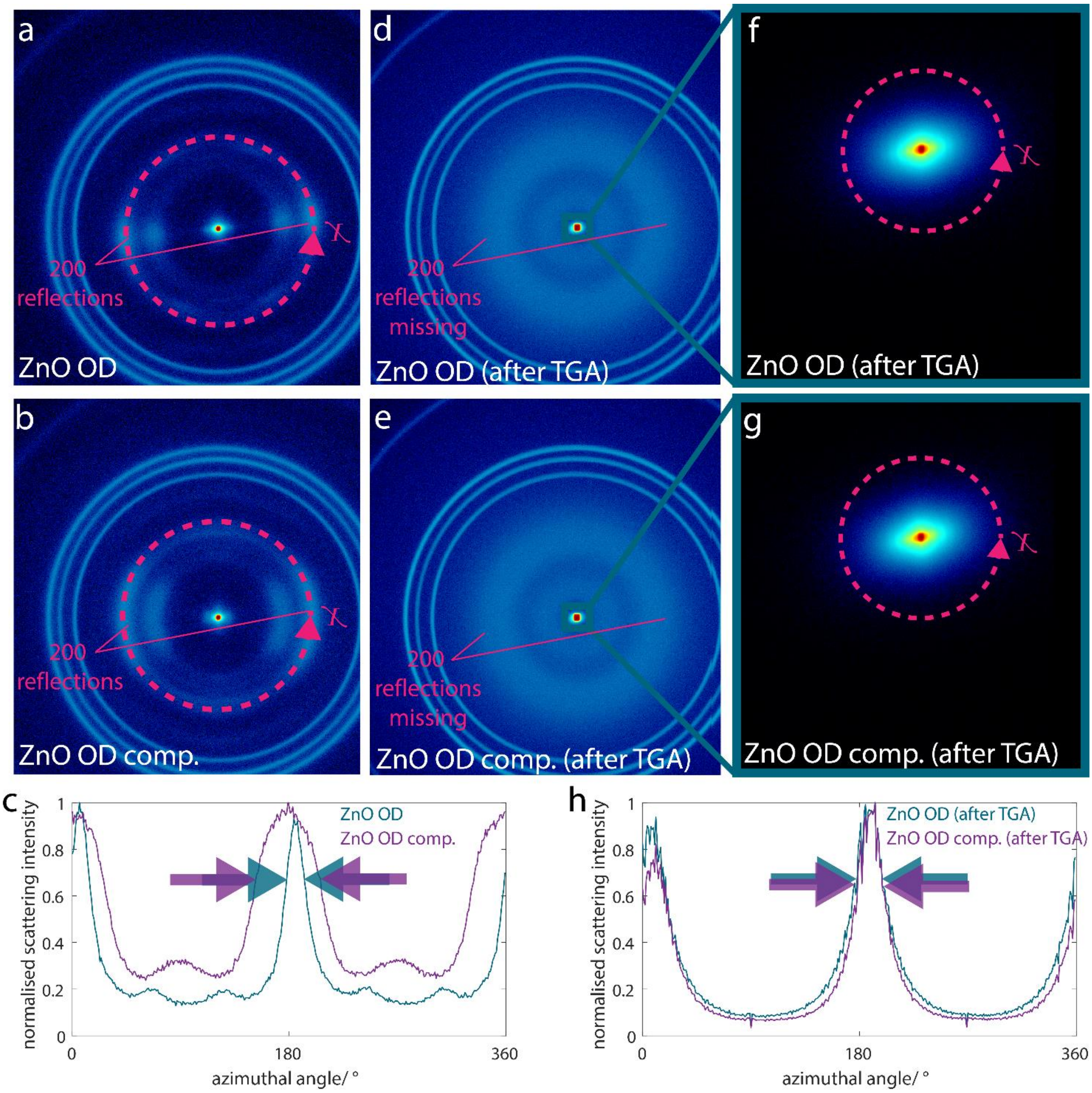

Figure S8. WAXD 2D signal of *ZnO OD* (**a**) and *ZnO OD* made from spruce compression wood (*ZnO OD comp.*) (**b**). The dotted line indicates the approx. position from where the azimuthal profiles (**c**) were obtained. The 200-reflection of *ZnO OD comp.* are broadened, indicating a larger MFA. WAXD 2D signal of ZnO-wood replicas obtained *ZnO OD* (**d**) and *ZnO OD comp.* (**e**). Due to the lack of 200-reflections no information on the MFA can be obtained from this signal. SAXS 2D signal of ZnO-wood replicas obtained *ZnO OD* (**f**) and *ZnO OD comp.* (**g**). The dotted line indicates the approx. position from where the azimuthal profiles (**h**) were obtained. No significant difference between the two azimuthal profile can be observed.

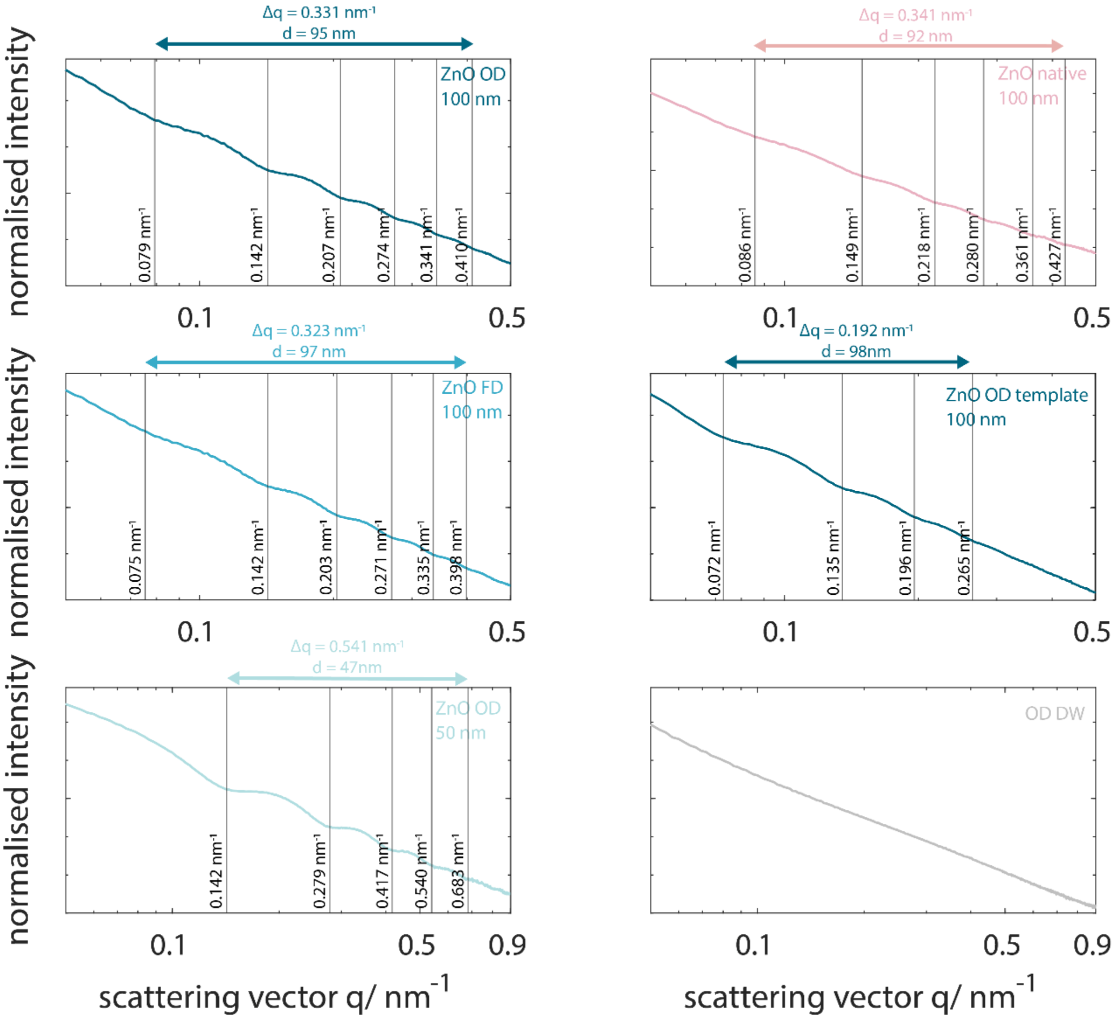

Figure S9. Detailed plot of the SAXS data obtained for determining the layer thickness of the ZnO inside the ZnO-wood hybrids. The layer thickness was calculated based on the equation $d_{ZnOlayer} = \frac{2\pi}{(\frac{q_n}{n})}$, where $d_{ZnO\ layer}$ is the ZnO layer thickness, q_n is the distance between n minima in the scattering pattern.

Table S2: Summary of the conductivity values for the different ZnO-wood hybrids along different directions.

***ZnO OD* 100 nm**			***ZnO OD* 50 nm**		
long/ Ωm	tang/ Ωm	rad/ Ωm	long/ Ωm	tang/ Ωm	rad/ Ωm
6 ± 3	38 ± 38	46 ± 45	35 ± 18	17 ± 8	17 ± 11
***ZnO FD* 100 nm**			***ZnO native* 100 nm**		
long/ Ωm	-	-	long/ Ωm	tang/ Ωm	rad/ Ωm
11 ± 4	-	-	2122 ± 1956	855 ± 645	5025 ± 4275

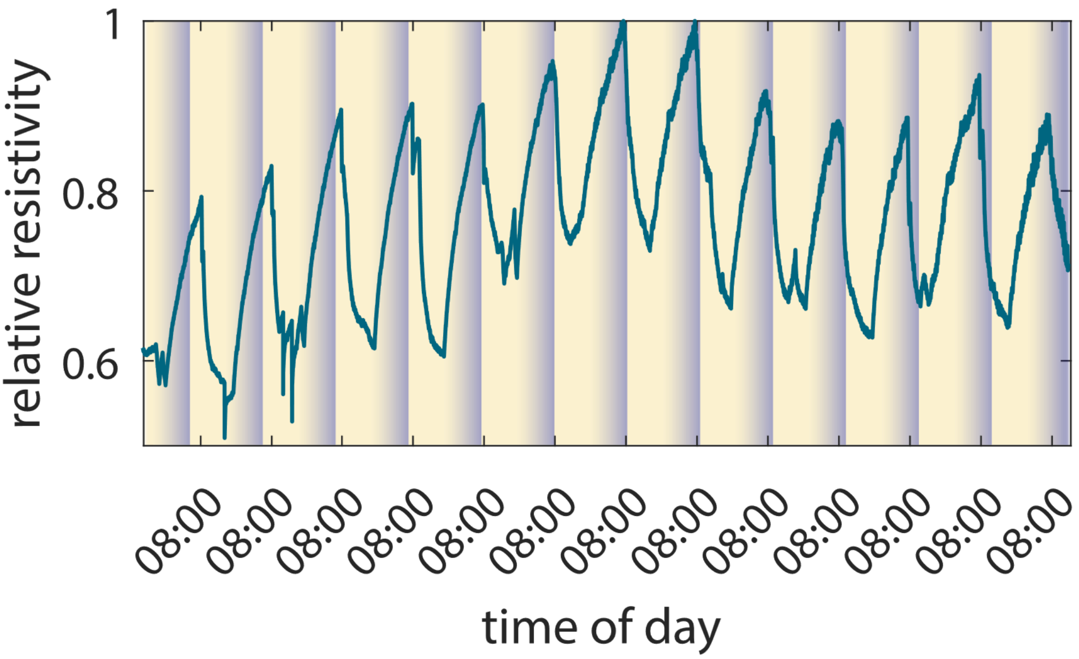

Figure S10. Measuring the resistivity of the specimen when exposed to naturally occurring exposure changes led to a clearly trackable day-night cycle.

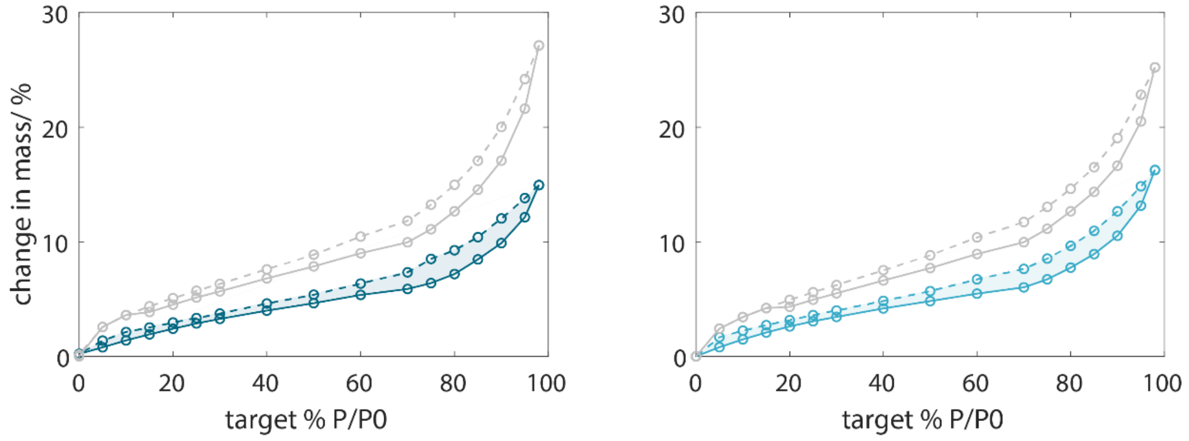

Figure S11. DVS sorption (*full lines*) and desorption (*dotted lines*) curves of *ZnO OD* and *DW OD* (*left*) and *ZnO FD* and *DW FD* (*right*).

Table S3. Summary of BET and dG specific surface areas determined by DVS.

	DW OD	***DW FD***	***ZnO OD***	***ZnO FD***
BET specific surface/ m^2g^{-1}	178	175	122	123
dG specific surface/ m^2g^{-1}	134	142	111	112

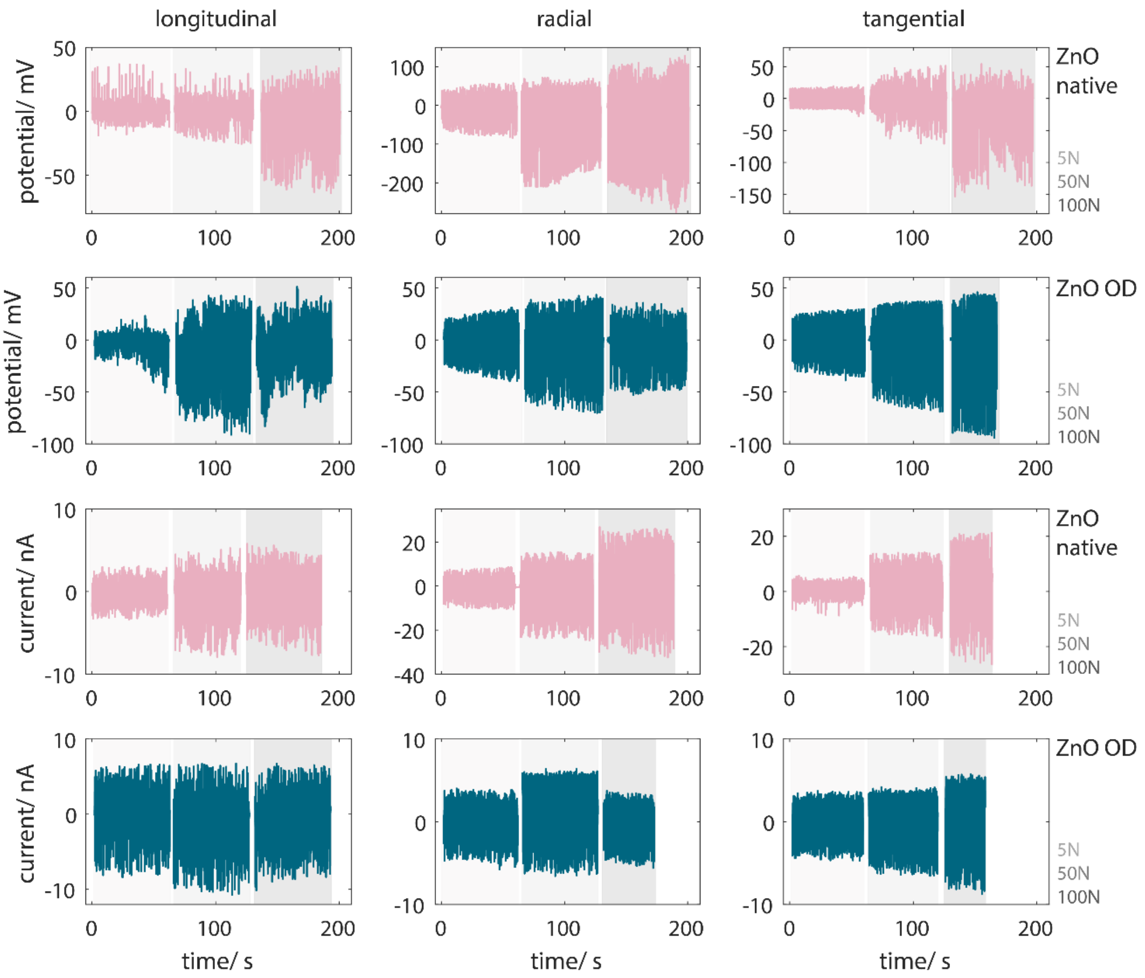

Figure S12. Piezoelectric response (potential and current) of *ZnO native* and *ZnO OD* tested at 5, 50 and 100 N in longitudinal, radial and tangential direction.

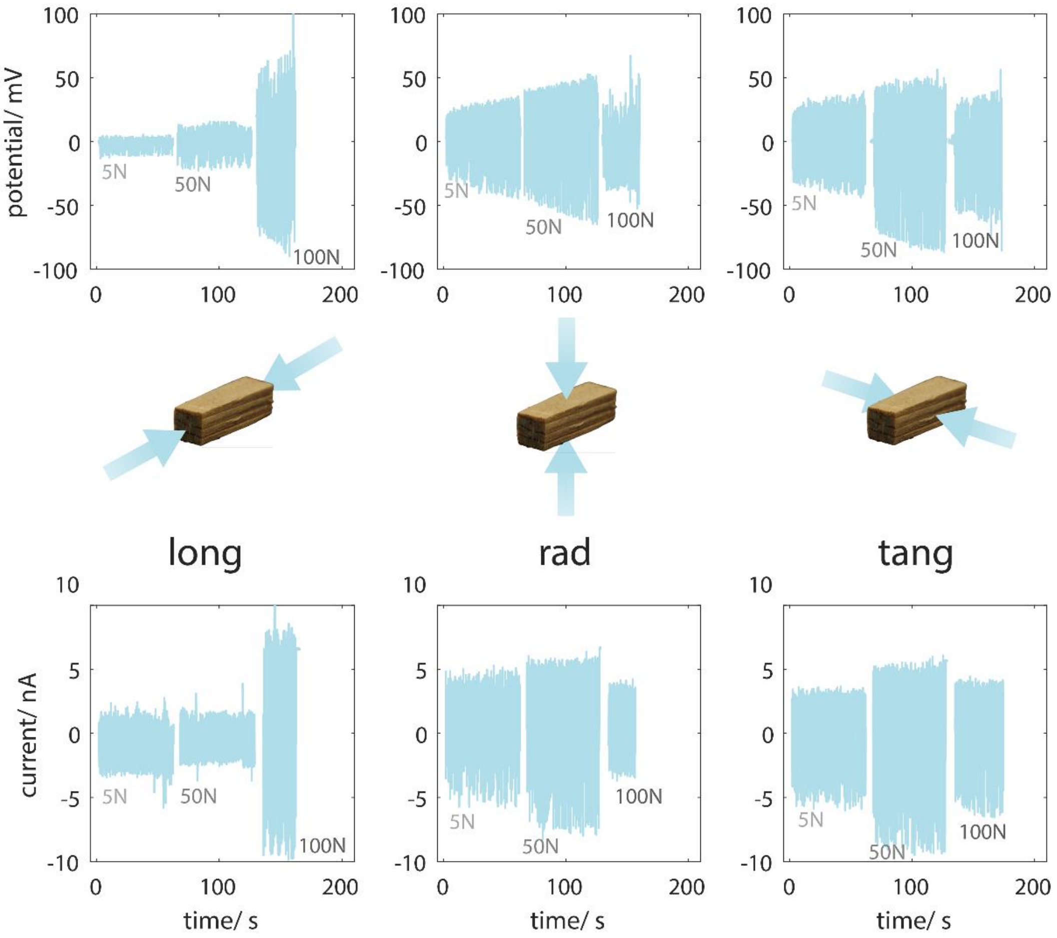

Figure S13. Piezoelectric response (potential and current) and *ZnO OD 50 nm* tested at 5, 50 and 100 N in longitudinal, radial and tangential direction.
